# Alleviation of water stress in soybean symbiosis by salicylic acid and methyl jasmonate-activated *Bradyrhizobium*

**DOI:** 10.1186/s12870-025-06806-1

**Published:** 2025-07-03

**Authors:** Tetiana Nyzhnyk, Edyta Kiedrzyńska, Sergii Kots, Maciej Zalewski, Marcin Kiedrzyński

**Affiliations:** 1 Institute of Plant Physiology and Genetics, Department of Symbiotic Nitrogen Fixation, National Academy of Sciences of Ukraine, Vasylkivska 31/17, Kyiv, 03022 Ukraine; 2 European Regional Centre for Ecohydrology of the Polish Academy of Sciences, Tylna 3, Lodz, 90-364 Poland; 3 Faculty of Biology and Environmental Protection, UNESCO Chair on Ecohydrology and Applied Ecology, University of Lodz, Banacha 12/16, Lodz, 90-237 Poland; 4 Faculty of Biology and Environmental Protection, Department of Biogeography, Paleoecology and Nature Conservation, University of Lodz, Banacha 1/3, Lodz, 90-237 Poland

**Keywords:** Hydrogen peroxide, Catalase, Superoxide dismutase, Nodulation, Nitrogen fixation, Growth regulators, *Glycine max* (L.) Merr

## Abstract

**Background:**

The use of exogenous compounds with growth-regulatory properties can play an effective part in providing plants with the necessary plastic resources for the synthesis of protective compounds. The aim of the research is to determine the effectiveness of salicylic acid and methyl jasmonate treatment for inducing resistance in soybean-*Bradyrhizobium* symbiosis, and improving symbiotic capacity.

**Methods:**

Soybean nodule bacteria *Bradyrhizobium japonicum* cultures were treated with salicylic acid (50 µM) or methyl jasmonate (0.75 µM) and used to create symbioses with soybean. The symbioses were cultivated under normal watering and water stress conditions, and the resulting plants were tested for *inter alia* pro-oxidant-antioxidant status, productivity and N-fixation activity.

**Results:**

The 0.75 µM methyl jasmonate treatment demonstrated 54.7% catalase and 14.6% greater superoxide dismutase (by 14.6%) activity, as well as and induced two-fold higher hydrogen peroxide, under water stress; in addition, nodulation processes were stimulated by 40% and inhibited nitrogen-fixing activity inhibited by 73.5%. The 50 µM salicylic acid treatment exhibited 54.5% lowered hydrogen peroxide, but 20.7% greater superoxide dismutase activity and 44.8% higher catalase activation water stress; this increased the efficiency of molecular nitrogen fixation (by 23.5%) and productivity (by 15.9%) in soybeans.

**Conclusions:**

Effect of 50 µM SA-treated *Bradyrhizobium* effectively activates the protective antioxidant mechanisms of soybean, improving symbiotic capacity and stress tolerance. Methyl jasmonate 0.75 µM in combination with *Bradyrhizobium* stimulates nodulation and inhibites nitrogen fixation in soybean under both optimal and insufficient water supply.

**Supplementary Information:**

The online version contains supplementary material available at 10.1186/s12870-025-06806-1.

## Introduction

The pursuit of ecohydrological solutions in the field of ecological biotechnologies contributes to the implementation of the United Nations Sustainable Development Goals (SDGs), particularly those related to water security, climate resilience, and sustainable agriculture. These actions are equally important in the agricultural sector, where innovative agroecological strategies are needed to improve the adaptability and water stress resilience of crop plants.

One of the main tasks of sustainable agricultural development is to identify new agroecological solutions to increase the adaptive properties and stress resistance of cultivated plants. Plant growth and development are regulated by the phytohormonal system, which participates in all processes of plant life, from seed germination to the formation of plant productivity [1, 2]. Phytohormones play an important role in plant responses to internal and external factors. One such group are the *stress phytohormones*, such as abscisic acid, ethylene, brassinosteroids, salicylates and jasmonates; many of these are used in plant treatments to mitigate the effects of stress factors [3, 4]. Complex functional interactions occur between these phytohormones, the mechanisms of which are still not fully understood.

Plant metabolism is regulated by a large number of synthetic and natural analogues of phytohormones together with various antioxidants and growth regulators (GRs) [5–7]. Among these, increasing attention has been drawn by jasmonates and salicylates; which are primarily used to protect plants from pathogenic microorganisms; many studies indicate them to be effective agents for increasing the resistance of plants to various extreme abiotic factors [1, 8, 9].

Salicylic acid, jasmonic acid and ethylene play important parts in the plant immune response, and their transduction pathways are associated with cytokinins, auxins and signals, as well as with DELLA protein activity [3, 4]. In addition, the ethylene, salicylic and jasmonic acids transduction pathways also interact with each other, modulating the plant response to stress factors and leading to the formation of systemically acquired resistance (SAR) [6, 10, 11].

Salicylic acid (SA) is believed to combines the properties of a signaling molecule and a phytohormone [12]. In addition to acting as a primary signal, it is also able to bind to the receptor proteins of the plasmalemma, thus activating the appropriate signaling systems to enable the synthesis of protective compounds and the formation of plant SAR to adverse factors [13–15]. It is also one of the mediators of the NADP oxidase and NO synthase signaling systems, both of which are known to play a role in reprogramming protein synthesis [6, 10]. It has been found that SA induces the synthesis of *pathogenesis-related* (PR) proteins at the transcriptional level. Regions containing SA-sensitive cis-active elements have been identified in the promoter regions of PR-protein genes, indicating that SA plays an important role in the expression of genes influencing the local and systemic resistance of plants [16, 17].

Salicylic acid is believed to play an important role in the in the formation the legume-rhizobial symbiosis, while preventing nodulation. To penetrate into the plant and successfully establish a symbiotic relationship with it, complementary rhizobia must first overcome various plant defense mechanisms, which are dependent on SA; it is believed they achieve this via Nod factor signalling [15]. Treatment of roots with a 0.1 mM SA solution has been found to completely inhibit the formation of indeterminate nodules and the mitogenic effect induced by Nod factors in pea (*Pisum sativum*), alfalfa (*Medicogo truncatula*), creeping clover (*Trifolium repens*); however, such treatment did not affect the formation of determinate nodules in beans (*Phaseolus vulgaris*), soybeans (*Glycine max*) or japanese licorice (*Lotus japonicus*) [7, 18].

Furthermore, inoculation of *Medicago sativa* with a complementary strain of *Rhizobium meliloti*, resulted in a decrease, or no change, in the level of SА in plant roots or no change. Conversely, inoculation of *Medicago sativa* with a strain of *Rhizobium leguminosarum* or a mutant of *Rhizobium meliloti* defective in Nod biosynthesis led to an accumulation of SА in the roots [13, 18]. Therefore, Nod factors synthesized by homologous rhizobia appear to be involved in suppressing SА-mediated defense systems in leguminous plants.

It has also been shown that SА-dependent protective signaling is rapidly activated at the early stages of symbiotic interaction (pre-infection), but is later suppressed, which promotes the growth of the infection thread in the root cortex and the formation of nodule primordia [19]. However, the molecular mechanisms mediating SА-induced protective signaling during nodulation are still largely unknown.

Jasmonic acid (JA) and its derivatives (jasmonates) are key regulators of plant development and their response to biotic and abiotic stresses. They support the development of the root and reproductive system of plants, and participate in the regulation of antioxidant metabolism, osmolyte synthesis, and metabolite accumulation [20]. In addition, JA interacts with other phytohormones through a complex signaling cascade to balance plant growth and development under stress. It has been found to have synergistic and antagonistic effects with other plant hormones, such as abscisic acid, ethylene and SA, in resisting stress factors [21]. Similarly, exogenous application of JA or its derivative methyl jasmonate (MJ) also has a regulatory effect on plants [9].

Although studies have addressed the complex nature of jasmonates and their active involvement in mitigating stressors, their underlying mechanism of action and biosynthetic pathways remain poorly understood, particularly the transduction of the jasmonate signal to the genetic apparatus of the cell. However, this pathway is believed to be based around specific proteins, in particular, the COI1 (coronative insensitive 1) protein, which is necessary for the removal of repressor proteins inhibiting of the transcription factors of JA signaling genes. In addition, a number of JA effects require the participation of reactive oxygen species (ROS) dependent mechanisms [9]. Nevertheless, the effect of ROS and their signaling pathways on the role of jasmonates remains poorly understood, as do those of other phytohormones in the transduction of symbiotic signals during the formation of legume-rhizobial symbioses.

Jasmonic acid and its derivatives, in particularly methyl jasmonate (MJ) can have both positive and negative effects with their influence on nodulation and nitrogen fixation processes depending on the type of legume, concentration of JA, and method of phytohormone application [22]. However, it remains unclear whether JA influences nodulation via the plant or the rhizobia, or a combination of both [18]. Therefore, additional research is needed to clearly clarify the role of JA in establishing symbiotic relationships.

Phytohormones play an important role in the activation of the main pathways of symbiotic signal transduction between macro- and microsymbionts; it is important to understanding their role and their integration with other metabolic pathways, particularly the pro-antioxidant systems, for increasing the symbiotic capacity and tolerance of the symbiosis during conditions of insufficient moisture.

The hypothesis of the study is that cultivating *Bradyrhizobium* with SA or MJ before addition to soybean will improve the functioning of the resulting symbiosis by optimizing plant metabolism and symbiotic capacity; the treatment will improve the regulation of pro-oxidant-antioxidant status status under conditions of water stress by inducing protective antioxidant mechanisms.

Such studies offer promise for increasing the efficiency of the soybean-*Bradyrhizobium* symbiosis under stress conditions by using growth regulators (GRs) as components of the bacterial cultivation medium; such treatment will support soybean symbiotic capacity and provide antioxidant protection for plants subjected to adverse environmental factors associated with climate change. Indeed, plant resistance inducers are cost-effective and environmentally beneficial strategies for protecting plants from stress factors.

The aim of the study is to determine the effect of SA or MJ treatments on the antioxidant properties of the resulting soybean-*Bradyrhizobium* symbiosis and its potential to mitigate water stress; the study also evaluates the effectiveness of complex inoculation for enhancing the realization of soybean symbiotic capacity.

It is assumed that such treatment will firstly, (i) regulate the pro-antioxidant status of plants; secondly, (ii) increase the symbiotic ability of soybean; and thirdly (iii) improve the productivity of the symbiosis in field conditions.

Our research is needed to intended to support the ecological direction of agricultural development and its transition to agroecological and organic means of production, taking into account such key factors as management and conservation of natural resources and adaptation to climate. Such activity is important to support rational agricultural management, taking into account innovative approaches to growing cultivated plants and, agroecological solutions, as essential components of sustainable agricultural development.

## Materials and methods

### Objects of research

Before sowing, soybean seeds (*Glycine max* (L.) Merr.) of the Almaz variety were inoculated with effective *Bradyrhizobium japonicum* B1-20. The inoculation was performed in culture medium supplemented with 50 µM SA (Bingospa, Poland) or 0.75 µM MJ (Fluorochem Ltd, Great Britain); these concentrations had been established experimentally. In the other experimental variants, the soybean seeds were inoculated with *B. japonicum* cultures without any growth regulator (GR) in the culture medium.

The soybean plants were of the Almaz variety-early maturing variety were selected for the study. Soybean seeds were kindly provided by Poltava State Agrarian Academy (Poltava, Ukraine). Originator–Poltava State Agrarian Academy, Ukraine. The variety from the 2007 Register of Plant Varieties of Ukraine and recommended for cultivation in the forest steppe of Ukraine.

An active, virulent Tn5 mutant of *Bradyrhizobium japonicum* B1-20 was created by transposon mutagenesis from the original strain 646 with the participation of *Esherichia coli* S17-1 with the plasmid pSUP5011, which contains the Tn5 transposon (pSUP5011::Tn5*mob*) at the Institute of Plant Physiology and Genetics of the National Academy of Sciences of Ukraine; deposited at the D.K. Zabolotny Institute of Microbiology and Virology of the National Academy of Sciences of Ukraine under registration number B-7538 [23].

### Cultivation of *Bradyrhizobium japonicum*

Cultivation of *Bradyrhizobium* was carried out in 750 mL flasks on a shaker (220 rpm) at a temperature of 26–28 °C. The organism was cultured on a liquid mannitol-yeast medium with the following composition (g/l): KH_2_PO_4_– 0.5, MgSO_4_– 0.2, NaCl– 0.1, yeast extract– 1.0, mannitol– 10.0. *Bradyrhizobium* cultures were taken in the exponential phase of growth (92–96 h) and used as seed material, with 2% of the volume of the medium introduced into the flasks as inoculum. The amount of inoculum that was introduced into the flasks was 2% of the volume of the medium. The number of *Bradyrhizobium* in the suspension added to 200 mL of nutrient medium was 10^8^ cells/ml. The purity of bacterial cultures was checked by sowing them on meat-peptone agar.

**Fig. 1:**
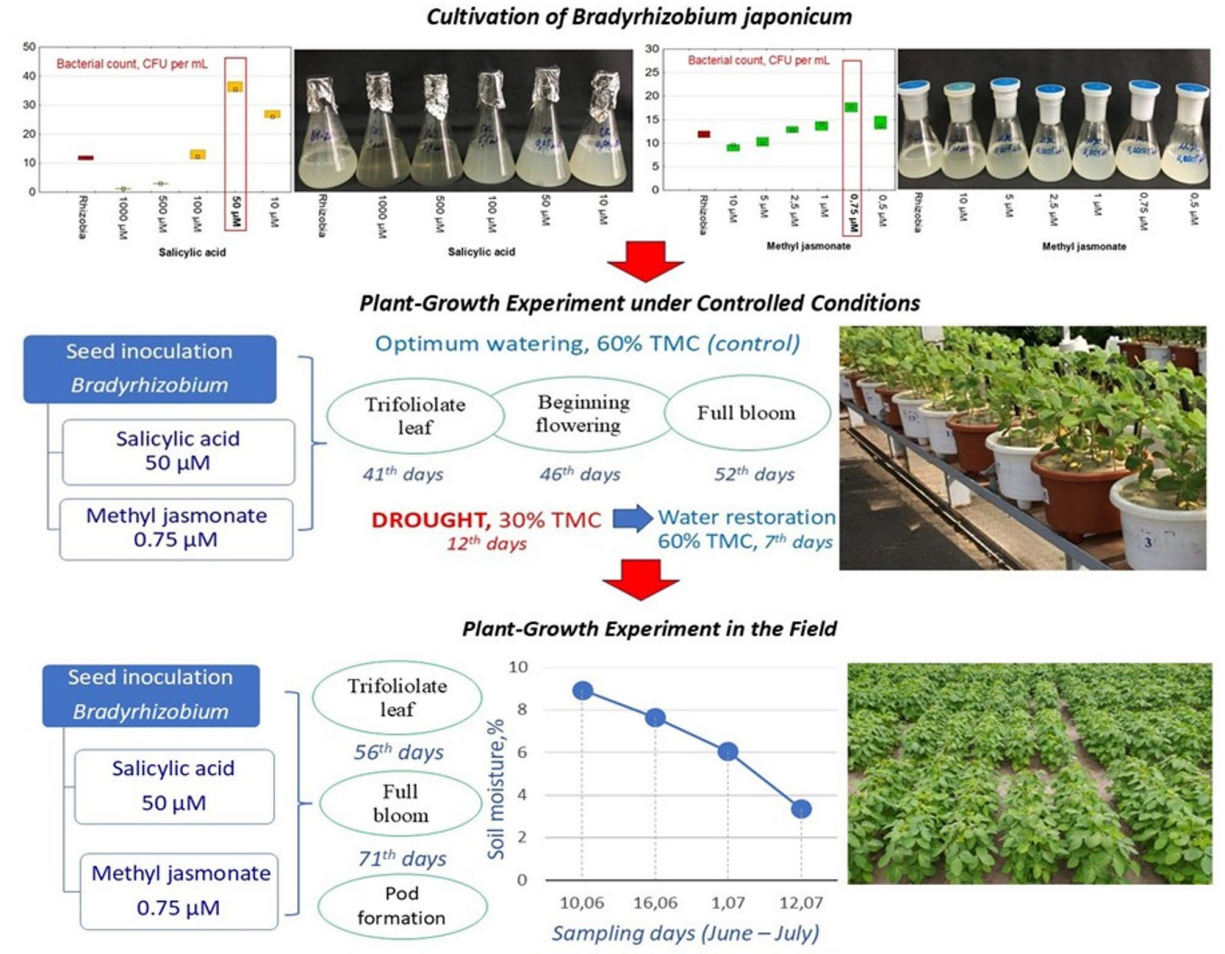
Schematic representation of the research stages: 1 - laboratory microbiological experiments to identify the most effective concentrations of SА/MJ in the *Bradyrhizobium* culture medium; 2 - controlled conditions with the creation of model water stress; 3 - field plots in uncontrolled conditions. TMC– total moisture capacity

The chosen concentrations of SА and MJ were selected based on previous experiments in which SA and MJ were added to the *Bradyrhizobium* cultivation medium (Fig. 1). Based on the findings, the most effective concentrations of SA and MJ for bacterial culture growth were selected (Fig. 1).

### Plant-growth experiment under controlled conditions

The plants were grown under strictly-controlled conditions in 10 kg vessels with a sterile substrate (sand) supplemented with Herligel nutrient mixture with a nitrogen rate of 0.25 (e.g. one quarter of the standard nitrogen treatment); the plants were cultivated under natural lighting and optimal moisture supply, i.e. 60% of total moisture capacity (TMC).

Soybean plants inoculated with bacteria were grown under natural light and optimal water supply, which was maintained at 60% total moisture content (TMС) by controlled watering. The amount of watering the plants was calculated based on the weight of the empty pot, as well as the dry weight of sand (per pot) and its moisture content. Before filling the pots, sterile sand was analyzed for moisture content (3.12%) and moisture capacity (19.67%) [24]; this was taken into account when creating different modes of watering plants.

The period of active nitrogen fixation by soybean was characterised by three stages: trifoliolate (40 days after sowing), budding-beginning flowering (47 days after sowing) and full bloom (52 days after sowing). The plants were subjected to water stress (WS) for a 12-day period comprising the five days of the trifoliolate stage (moderate WS) continuing for seven days into the budding-beginning flowering stage (prolonged WS). During this period, control plants received 60% TMC - optimal water supply (control). After this period, i.e. in the full bloom stage of soybean, the stressed plants were provided optimal watering (60% TMC) for a seven-day restoration period (Fig. 1).

To carry out the research, the soybean nodules were selected at the trifoliolate leaf stage, at the beginning of flowering, and full bloom. Soybean plants inoculated with *Bradyrhizobium* that had not received SA or MJ treatment were used as controls.

### Plant-growth experiment in the field

Soybean sowing was carried out in early May. The seeds were sown to a depth of 3–5 cm at the rate of 40 seeds per one linear meter, when the upper sowing layer of soil was 10–15°С (Fig. 1). Plots were placed randomly. Their accounting area was 5 m^2^. Repetition was four-fold. During the vegetative period, the vegetative mass of plants was measured. At the trifoliolate leaf, full bloom and pod formation stages, plants were selected to determine the nodulation ability of *B. japonicum* (number, mass of nodules, NFA) in 10-fold repetition. Samples of nodules for analysis were taken in stages: trifoliolate leaves, full bloom and pod formation. Plants inoculated with *Bradyrhizobium* without GRs used as controls.

### Moisture content

Relative humidity was determined using the thermo-gravimetric method. Sand samples were taken in aluminum boxes and covered with lids. They were transported to the laboratory and weighed on electric scales with an accuracy of 0.1 g. After weighing, the samples were dried to a constant mass in boxes with open lids at a temperature of 100–105 °C, which allowed all moisture to be removed from the sand. The moisture content of the sand (% dry weight (DW)) was calculated from the difference in the mass of the sample before and after drying [24].

### Moisture capacity

A known mass of sand was placed in glass cylinders. Each such cylinder contained a hole at the bottom, which was closed by a filter disc. The cylinder was then closed from above with a glass stopper and placed in a desiccator with water, so that the sand was completely saturated with water through the hole in the cylinder. After 24 and 48 h, the cylinders with the wet sand were weighed. The relative humidity of the sand was calculated from the difference in the mass of the sample before and after saturation with water (in percent) [24].

### Content of hydrogen peroxide

Hydrogen peroxide (H_2_O_2_) content was determined by the ferrothiocyanite method [25]. The plant material was treated with a cooled solution of 5% trichloroacetic acid at a ratio of 1:3 (weight / volume). The supernatant was obtained by centrifugation at 14,000 rpm for five minutes (4ºС). The peroxide concentration was determined spectrophotometrically at a wavelength of 480 nm following a color reaction with potassium thiocyanate; the result was compared against a calibration curve. The results are presented in µmol per gram of dry weight (DW). The mass of dry matter was determined by drying the samples to a constant value at a temperature of + 105 ºС.

### Extraction and determination of superoxide dismutase activity

To obtain the enzyme extract, a portion of the plant material was triturated in a ratio of 1:2 (weight / volume) with chilled 0.5 M Tris-HCl buffer (pH 7.8), which contained which contained 2 mM EDTA, 1 mM PMSF, 5 mM β-mercaptoethanol and 1% polyvinylpyrrolidone. The homogenate was centrifuged at 10,000 rpm for 20 min at 4ºС. The supernatant was used to determine enzyme activity. The activity of superoxide dismutase (SOD) (EC 1.15.1.1) was determined by its ability to inhibit the photochemical reduction of nitroblue tetrazolium [26]. The reaction mixture contained 50 mM phosphate buffer (pH 7.8), 13 mM methionine, 2 µM riboflavin, 63 µM p-nitroblue tetrazolium, 0.1 mM EDTA, and 100 µL enzyme extract. The reaction proceeded for 15 min at an illumination intensity of 70 µmol quanta/(m2 · s) when illuminated by fluorescent lamps with a power of 15 W. The optical density was measured at 560 nm. Results are presented in units of enzyme activity (U) per mg of protein in the supernatant.

### Extraction and determination of catalase activity

To obtain the enzyme extract, part of the plant material was triturated (ratio 1:2) with cooled 0.5 M Tris-HCl buffer (pH 7.8) containing 5 mM β-mercaptoethanol and 0.1% polyvinylpyrrolidone. The homogenate was centrifuged at 10,000 rpm (4ºС) for 20 min. The supernatant was collected and analyzed for catalase activity (CAT) (EC 1.11.1.6.) by developing a color reaction with ammonium molybdate; the concentration was measured at a wavelength of 410 nm [27]. Results are presented in units of enzyme activity (U) per mg of protein in the supernatant.

### Total soluble protein

Soluble protein content was measured based on the standard curve method using bovine serum albumin [28].

### Nodylation potential of *Bradyrhizobium japonicum*

To determine the nodulation potential of *Bradyrhizobium*, 20 typical plants of each experimental variant were selected and the roots were washed; then nodules were separated and the mean number and mass per plant were calculated. The results are presented as number (pcs) and mass (g) of nodules per plant.

### Nitrogen-fixing activity

Nitrogen fixation activity (NFA) was determined by the acetylene reduction method [29]. For this, the roots with nodules were placed in hermetically-sealed glass vials with a capacity of 75 cm^3^, in which a 10% concentration of acetylene was created. The samples were incubated for one hour. Following this, the gas mixture in the vial, which contained ethylene formed by the reduction of acetylene by nitrogenase, was analyzed using an Agilent GC system 6850 gas chromatograph (USA) with a flame ionization detector. Gas separation was carried out on a column (Supelco Porapak N) at a thermostat temperature of 55 °C and a detector temperature of 150 °C. The carrier gas was helium (20 mL per one min). The volume of the analyzed gas mixture sample was 1 cm^3^. Pure ethylene was used as a standard (Sigma-Aldrich, No. 536164, USA). NFA is presented in molar units of ethylene formed (µmol С_2_Н_4_) per plant per hour.

### Statistical data analysis

All statistical analyses were performed using the STATISTICA ver. 13.3 software package [30]. The Kruskal-Wallis test was used to compare the differences between the studied variants of *Bradyrhizobium*, and the SA and MJ treatments, for each set of growing conditions, viz. optimal irrigation and water stress. Significant differences are indicated by letters in the figures.

To analyze the physiological and biochemical responses of plants under varying experimental treatments and water conditions, were employed multidimensional scaling (MDS) and a scatterplot analysis to visualize relationships between treatments, variables, and experimental setups. The statistical analyses were conducted using the R language [31].

The MDS analysis was performed to reduce the dimensionality of the dataset and explore the relationships between experimental treatments based on the measured variables (Nod, NFA, SOD, and H₂O₂). Classical MDS was implemented using the *cmdscale()* function from the *stats* package [31]. Euclidean distance have been used. The percentage of variability explained by each dimension was calculated based on eigenvalues of the distance matrix. The visualization of the MDS results, including the addition of variable vectors, was created using the *ggplot2* package [32]. Arrows representing variable contributions to the dimensions were added based on correlations between the original variables and the MDS dimensions.

To directly explore the relationship between two key variables (Nod and NFA), we used a scatterplot approach. The data were preprocessed and subsetted using the *dplyr* package [33]. The scatterplot was generated with *ggplot2* [32]. The *scales* package [34] was employed to fine-tune axis labels and add percentage information for explained variability in the MDS plot.

## Results

### Pro-antioxidant status of soybean nodules

**Fig. 2:**
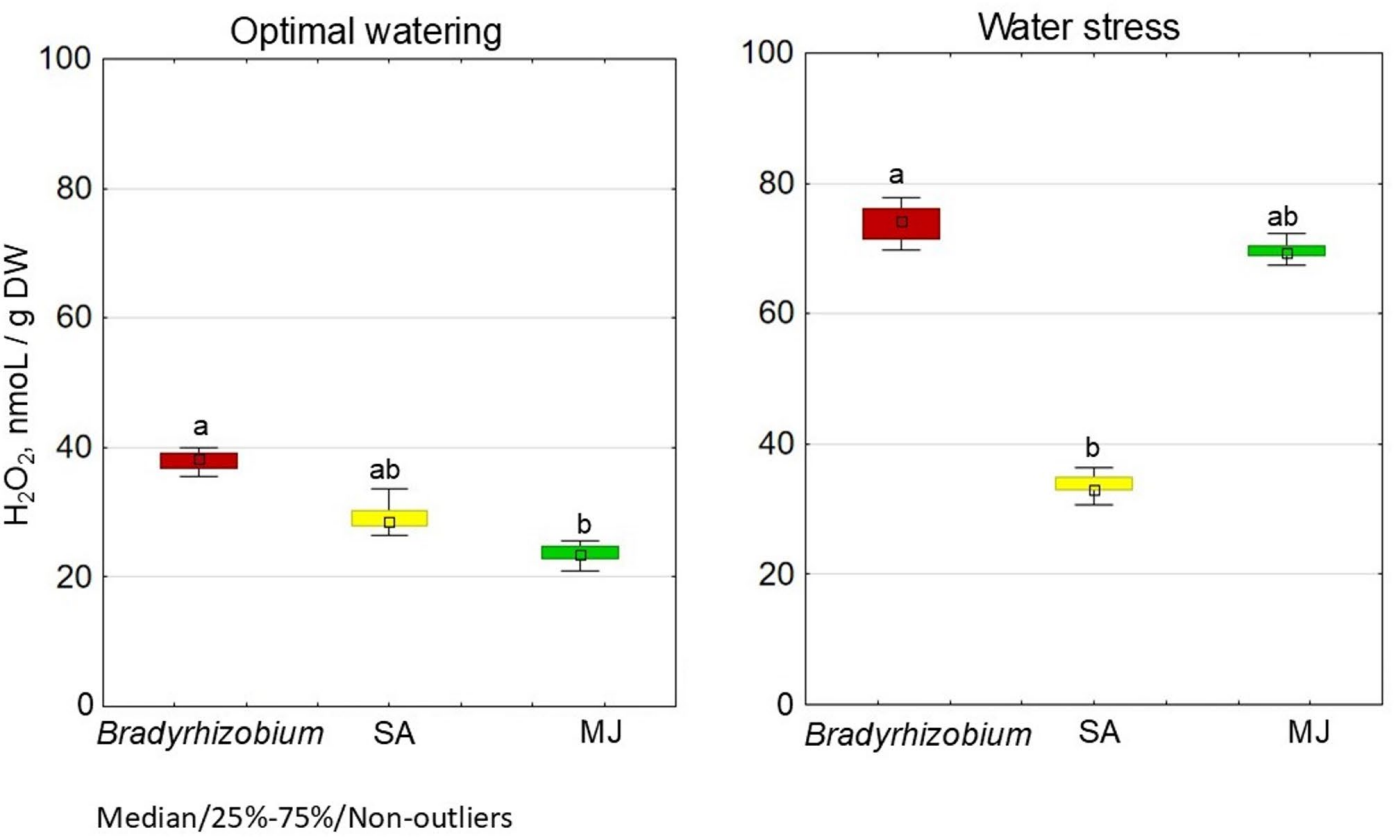
Effect of SA- and MJ-treated *Bradyrhizobium* on the H_2_O_2_ production in soybean nodules under optimal and water stress conditions. The figure shows the total value of the indicators analyzed during the trifoliolate leaf, budding-beginning flowering and full bloom stages; the letters indicate significant differences between the variants (*p* < 0.05); Kruskal-Wallis test. Experimental treatments, MJ: methyl jasmonate (green), SA: salicylic acid (yellow), *Bradyrhizobium*: control (red)

The addition of SА or MJ in the *Bradyrhizobium* cultivation medium led to a statistically significant decrease in H_2_O_2_ content in soybean nodules under optimal plant growth conditions. In addition, MJ treatment resulted in the lowest production in nodules (23.5 µmoL/g of DW) among the tested variants: H_2_O_2_ concentration decreased by 38.0% relative to controls for MJ, and by 22.6% for SA (Fig. 2).

Under conditions of water stress, the lowest H_2_O_2_ concentration in nodules (33.6 µmoL/g of DW) was recorded in soybeans inoculated with *Bradyrhizobium* containing SA; this value was 54.5% lower than controls, i.e. without GRs (Fig. 2). In the variant with MJ, the H_2_O_2_ level in nodules was almost the same as that controls.

**Fig. 3:**
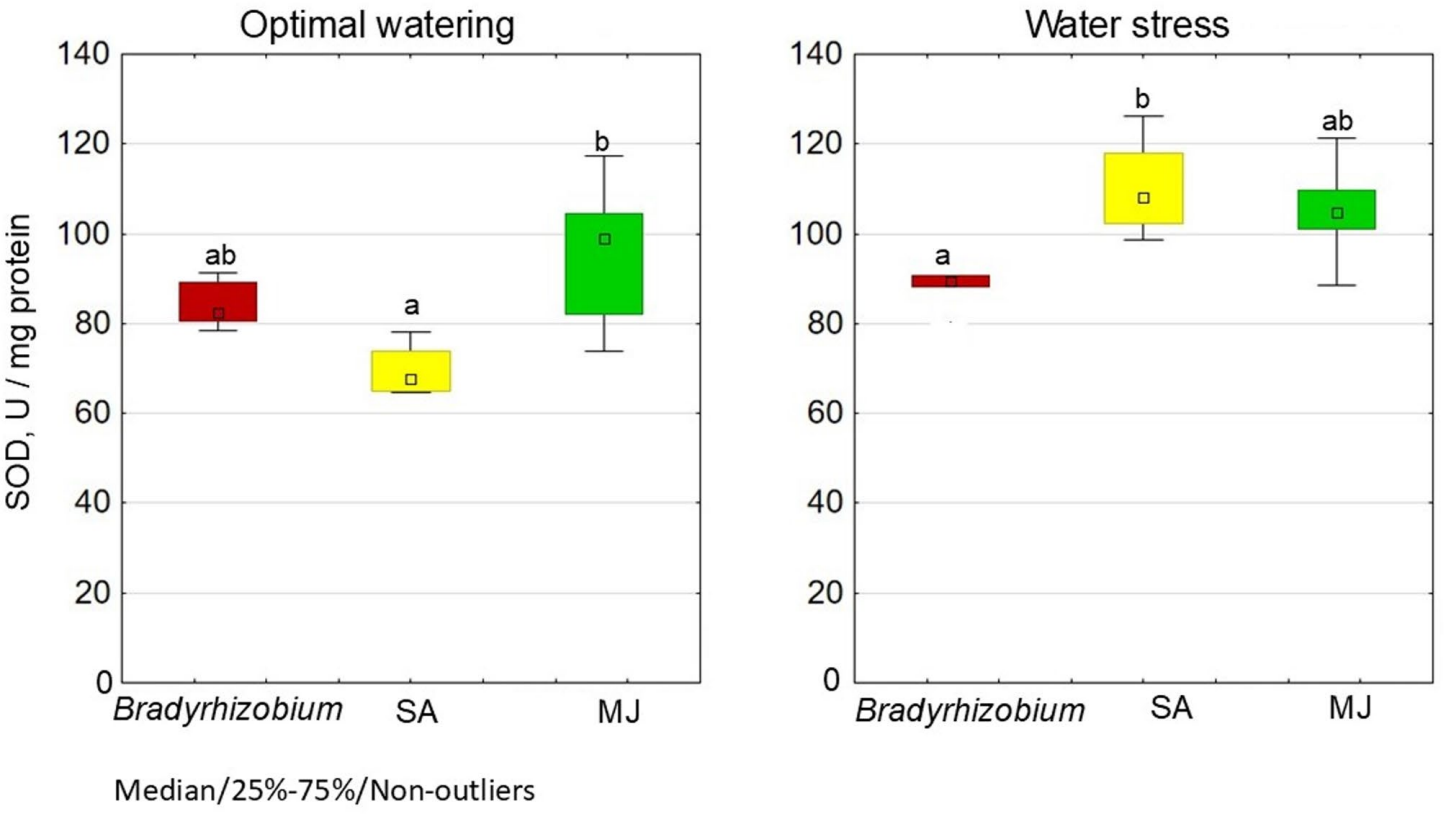
Effect of SA- and MJ-treated *Bradyrhizobium* on SOD activity in soybean nodules under optimal and water stress conditions. The figure shows the total value of the indicators analyzed during the trifoliolate leaf, budding-beginning flowering and full bloom stages; the letters indicate significant differences between the variants (*p* < 0.05); Kruskal-Wallis test. Experimental treatments, MJ: methyl jasmonate (green), SA: salicylic acid (yellow), *Bradyrhizobium*: control (red)

**Fig. 4:**
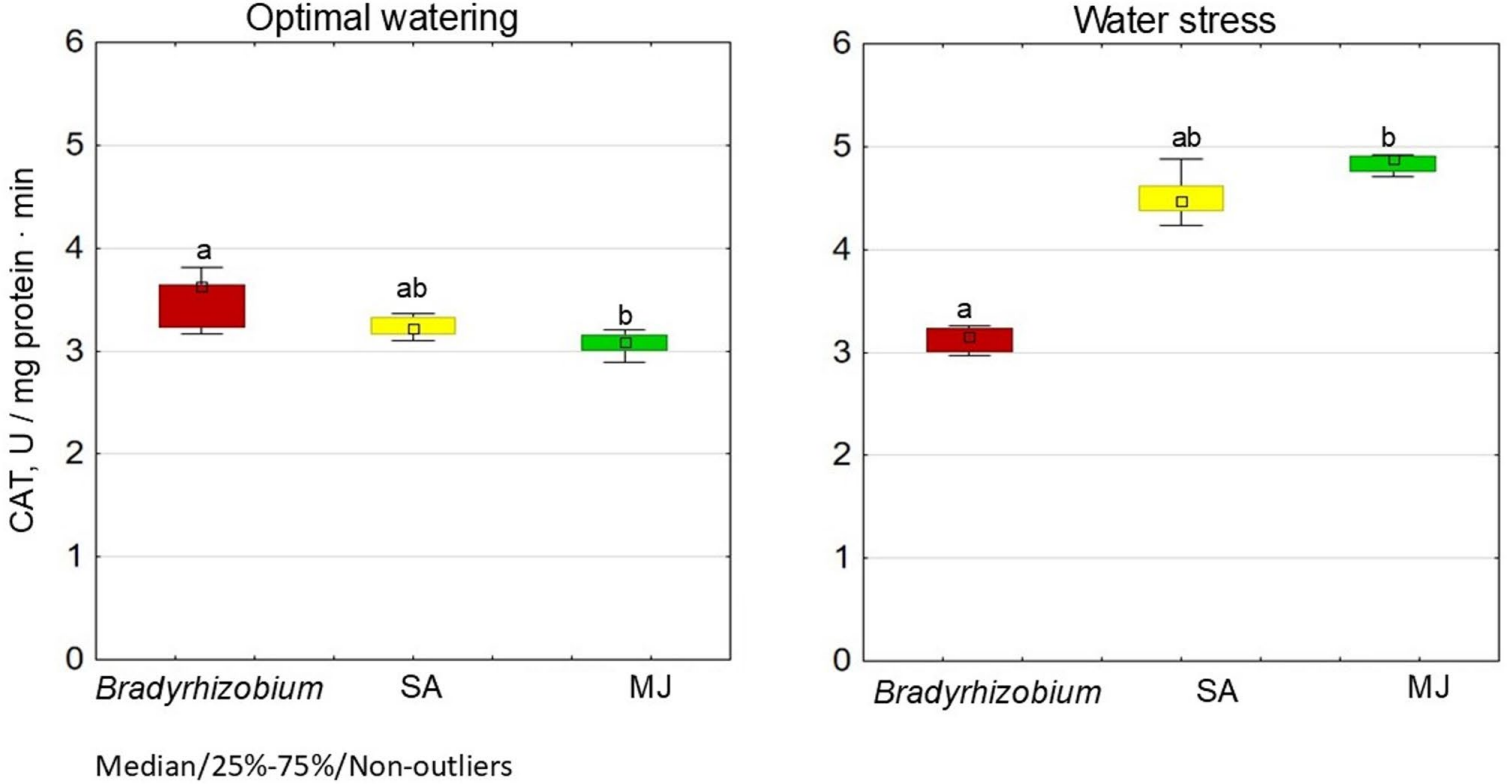
Effect of SA- and MJ-treated *Bradyrhizobium* on CAT activity in soybean nodules under optimal and water stress conditions. The figure shows the total value of the indicators analyzed during the trifoliolate leaf, budding-beginning flowering and full bloom stages; the letters indicate significant differences between the variants (*p* < 0.05); Kruskal-Wallis test. Experimental treatments, MJ: methyl jasmonate (green), SA: salicylic acid (yellow), *Bradyrhizobium*: control (red)

Of the three variants, the highest SOD activity (95.3 U/mg protein) and the lowest CAT activity (3.0 U/mg protein) in nodules under optimal water irrigation were recorded in the variant with MJ (Figs. 3 and 4).

Under these conditions, the SA variant demonstrated a decrease in SOD activity (69.8 U/mg protein), which was 17.2% lower than the controls (without GRs), and approximately the same, CAT activity as controls.

Under conditions of water stress, the variants with GR modification demonstrated 20.7% (SA) and 14.6% (MJ) increases in SOD activity relative to controls (Fig. 4). Both GR variants demonstrated higher CAT activity compared to SOD; the SA variant was 44.8% higher, and MJ was 54.7% higher (Fig. 4).

**Fig. 5:**
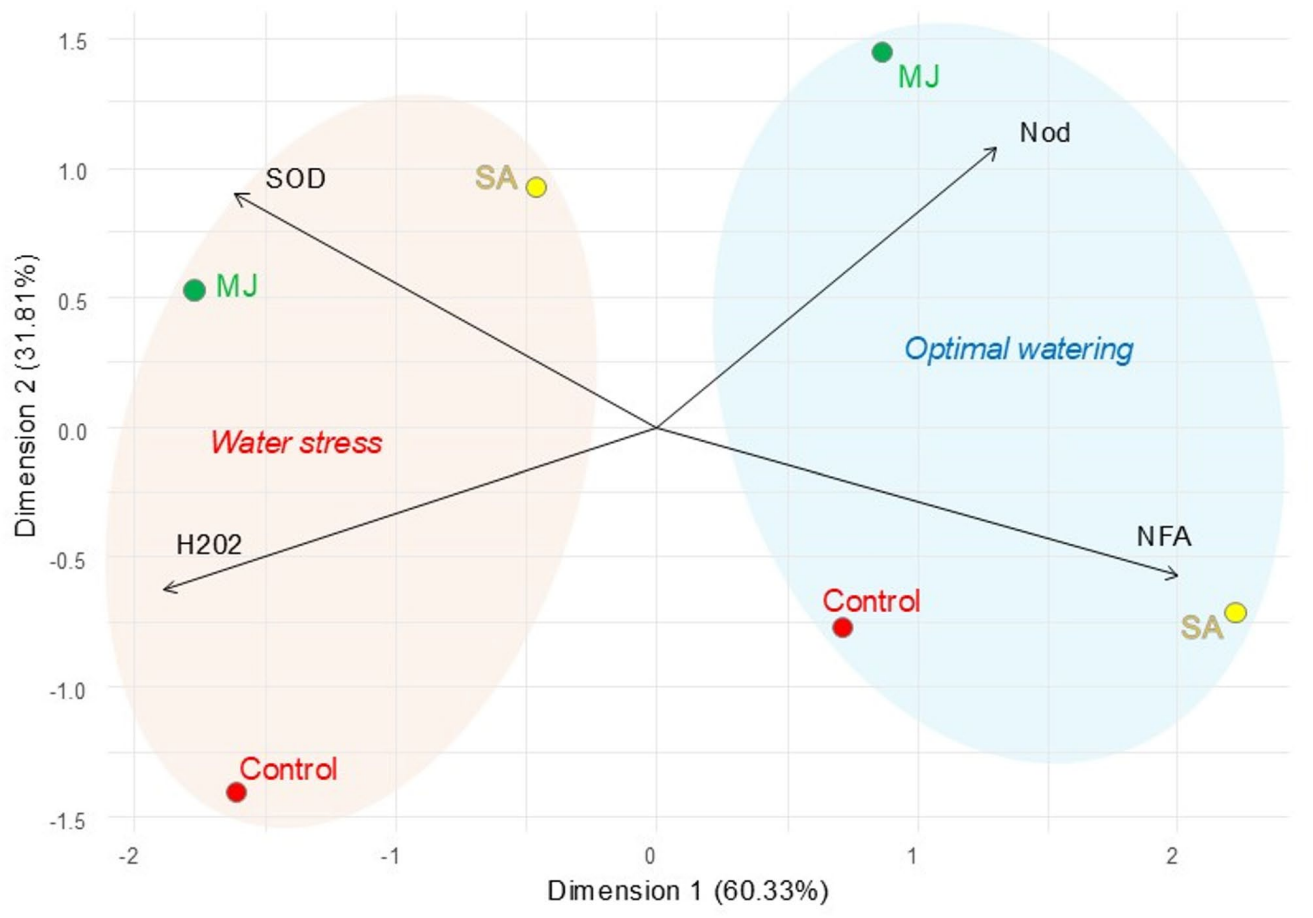
Multidimensional scaling (MDS) analysis of experimental treatments based on physiological and biochemical variables in soybean under optimal and water stress conditions. Arrows represent variable contributions in the analysis of parameters: SOD - superoxide dismutase, H_2_O_2_ - hydrogen peroxide, Nod - *Bradyrhizobium* nodulation potential, AFA - nitrogen-fixing activity. Experimental treatments, MJ: methyl jasmonate (green), SA: salicylic acid (yellow), *Bradyrhizobium*: control (red)

The multidimensional scaling (MDS) analysis revealed that the first two dimensions explained a cumulative 92.14% of the total variability in the dataset (Fig. 5). This indicates that the two-dimensional space sufficiently represents the relationships between experimental cases.

The experimental conditions formed distinct clusters in the MDS plot (Fig. 5). Cases associated with water stress are characterized by higher contributions from the variables SOD and H₂O₂, indicating their association with oxidative stress. In contrast, cases under optimal watering clustered are associated to strong contributions from Nod and NFA, highlighting enhanced nodulation and nitrogen fixation under favorable conditions. The vector orientations of these variables further supported their roles in distinguishing experimental conditions. Specifically, Nod and NFA were positively correlated with optimal watering, whereas SOD and H₂O₂ were strongly associated with water stress conditions.

The inclusion of experimental treatments — MJ and SA — reveals distinct clustering patterns under varying water conditions (Fig. 5). Under water stress conditions, variant with MJ is closely aligned with higher levels of SOD and H₂O₂, which are indicative of oxidative stress responses. Addition of SA in optimal watering conditions, show higher contributions from NFA, but MJ variant from Nod parameter.

### Soybean symbiotic capacity

**Fig. 6:**
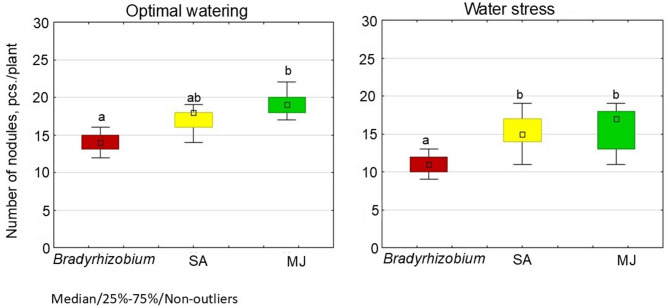
Effect of SA- and MJ-treated *Bradyrhizobium* on the nodulation in soybean under optimal and water stress conditions. The figure shows the total value of the indicators analyzed during the trifoliolate leaf, budding-beginning flowering and full bloom stages; the letters indicate significant differences between the variants (*p* < 0.05); Kruskal-Wallis test. Experimental treatments, MJ: methyl jasmonate (green), SA: salicylic acid (yellow), *Bradyrhizobium*: control (red)

**Fig. 7:**
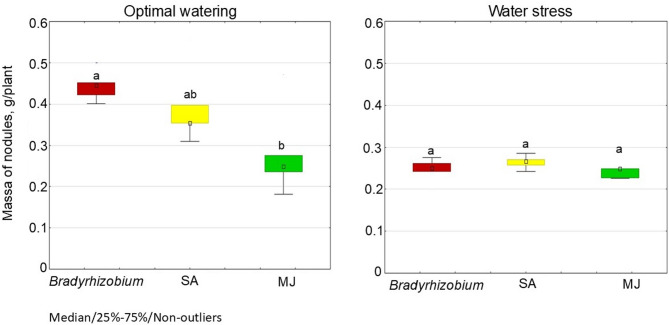
Effect of SA- and MJ-treated *Bradyrhizobium* on the mass of soybean nodules under optimal and water stress conditions. The figure shows the total value of the indicators analyzed during the trifoliolate leaf, budding-beginning flowering and full bloom stages; the letters indicate significant differences between the variants (*p* < 0.05); Kruskal-Wallis test. Experimental treatments, MJ: methyl jasmonate (green), SA: salicylic acid (yellow), *Bradyrhizobium*: control (red)

Under optimal water conditions, the addition of GRs to the bacterial culture media stimulated the nodulation potential of *Bradyrhizobium*. The highest number of nodules on soybean roots (19.2 pcs./plant) (Fig. 6) was recorded in the variant with MJ, which showed the lowest indicators by mass of nodules (0.28 g/plant), compared to other variants. A similar tendency was observed on soybean roots for SA, with the number of nodules increasing by 21.2%, compared to controls (Fig. 6). In addition, the SA variant yielded nodules with a greater mass (0.39 g/plant) than the MJ variant (Fig. 6). Both variants with GRs, demonstrated a similar tendency to stimulate nodulation under conditions of water stress. In particular, the indicators of the number of nodules increased by 40.0% in variants with SA and MJ, compared to control plants without GR (Fig. 6); however, there no significant difference in nodule mass index was observed between the studied variants (Fig. 7).

**Fig. 8:**
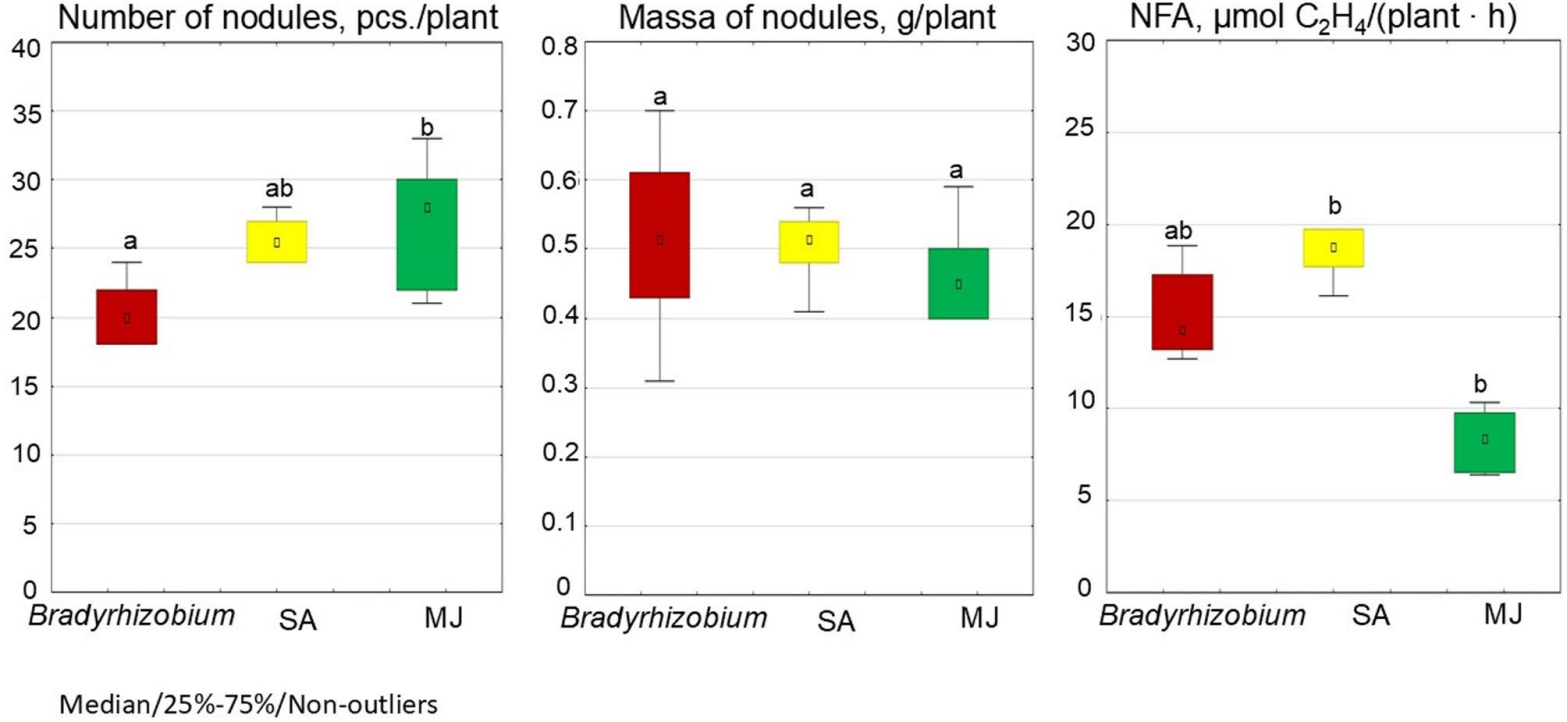
The effect of pre-sowing inoculation with SA- or MJ-treated *Bradyrhizobium* on nodulation and nitrogen fixation in soybeans in the plot field experiment. The figure shows the total value of the indicators analyzed during the trifoliolate leaf, budding-beginning flowering and full bloom stages; the letters indicate significant differences between the variants (*p* < 0.05); Kruskal-Wallis test. Experimental treatments, MJ: methyl jasmonate (green), SA: salicylic acid (yellow), *Bradyrhizobium*: control (red)

**Fig. 9:**
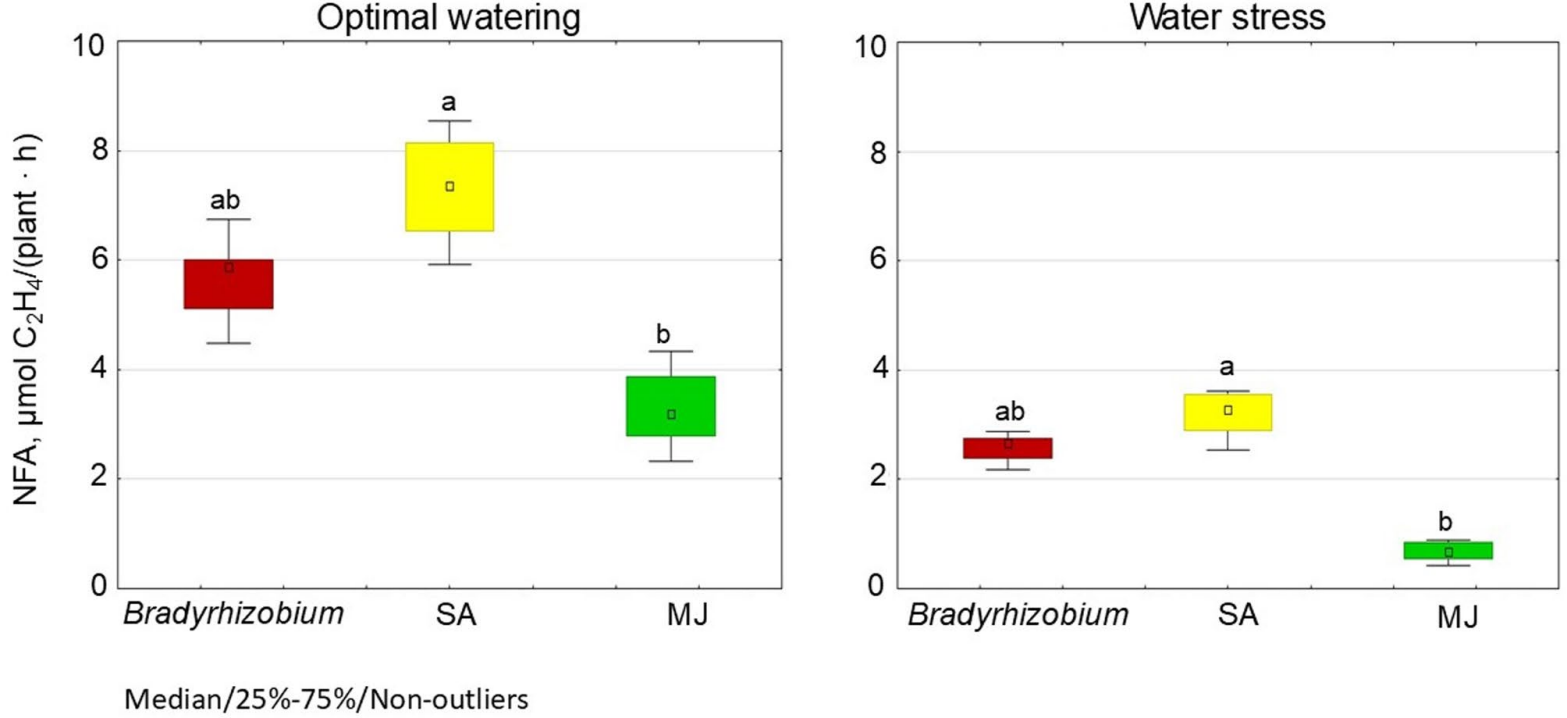
Effect of SA- and MJ-treated *Bradyrhizobium* on the NFA in soybean nodules under optimal and water stress conditions. The figure shows the total value of the indicators analyzed during the trifoliolate leaf, budding-beginning flowering and full bloom stages; the letters indicate significant differences between the variants (*p* < 0.05); Kruskal-Wallis test. Experimental treatments, MJ: methyl jasmonate (green), SA: salicylic acid (yellow), *Bradyrhizobium*: control (red)

Under optimal water supply, the MJ group demonstrated lower nitrogen fixation activity (2.3 µmoL С_2_Н_4_/plant · h) compared to controls, i.e. without GR; this value was 41.4% lower (Fig. 8). In contrast, the SA variant demonstrated the highest NFA (5.9 µmoL С_2_Н_4_/plant · h), this value being 29.2% higher than control values (Fig. 9).

A similar trend for NFA index was observed under water stress. In particular, the lowest NFA (0.4 µmoL С_2_Н_4_/plant · h) was recorded in the MJ variant, i.e. 73.5% lower than than controls (Fig. 8). In contrast, in the SA variant, the level was 23.5% higher than controls (Fig. 9).

The plants were then grown in uncontrolled conditions in a plot field experiment. It was found that pre-sowing inoculation of soybean seeds with *Bradyrhizobium* containing SA or MJ stimulated nodulation. The highest number of nodules was recorded in the MJ variant (28 pcs./plant), which exceeded the control level by 32.6% (Fig. 8). In comparison, the number of nodules in the SA variant was 26.2% higher than controls (Fig. 8). While GR appears to have a simulating effect on the number of nodules on soybean roots, no statistically significant difference in nodule mass was found between the investigated variants (Fig. 8).

In the plot field conditions, the highest efficiency of nitrogen fixation by soybean nodules was observed in the SA variant (18.5 µmoL С_2_Н_4_/plant · h), which was 22.4% higher than controls (i.e. without GR) (Fig. 8). The lowest level of nitrogen fixation activity (8.3 µmoL С_2_Н_4_/plant · h) was recorded in the MJ variant, which was 45.0% lower than controls (Fig. 8).

**Fig. 10:**
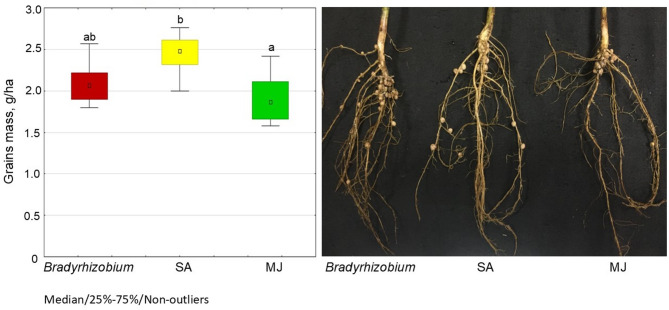
Productivity of soybeans under plot field conditions. The letters indicate significant differences between the variants (*p* < 0.05); Kruskal-Wallis test. Experimental treatments, MJ: methyl jasmonate (green), SA: salicylic acid (yellow), *Bradyrhizobium*: control (red)

Regarding soybean grain productivity, the SA variant demonstrated 15.9% higher grain mass than controls (i.e. without GR) (Fig. 10). However, no statistically significant differences in soybean grain yield were observed between the MJ variant and controls (Fig. 10).

**Fig. 11:**
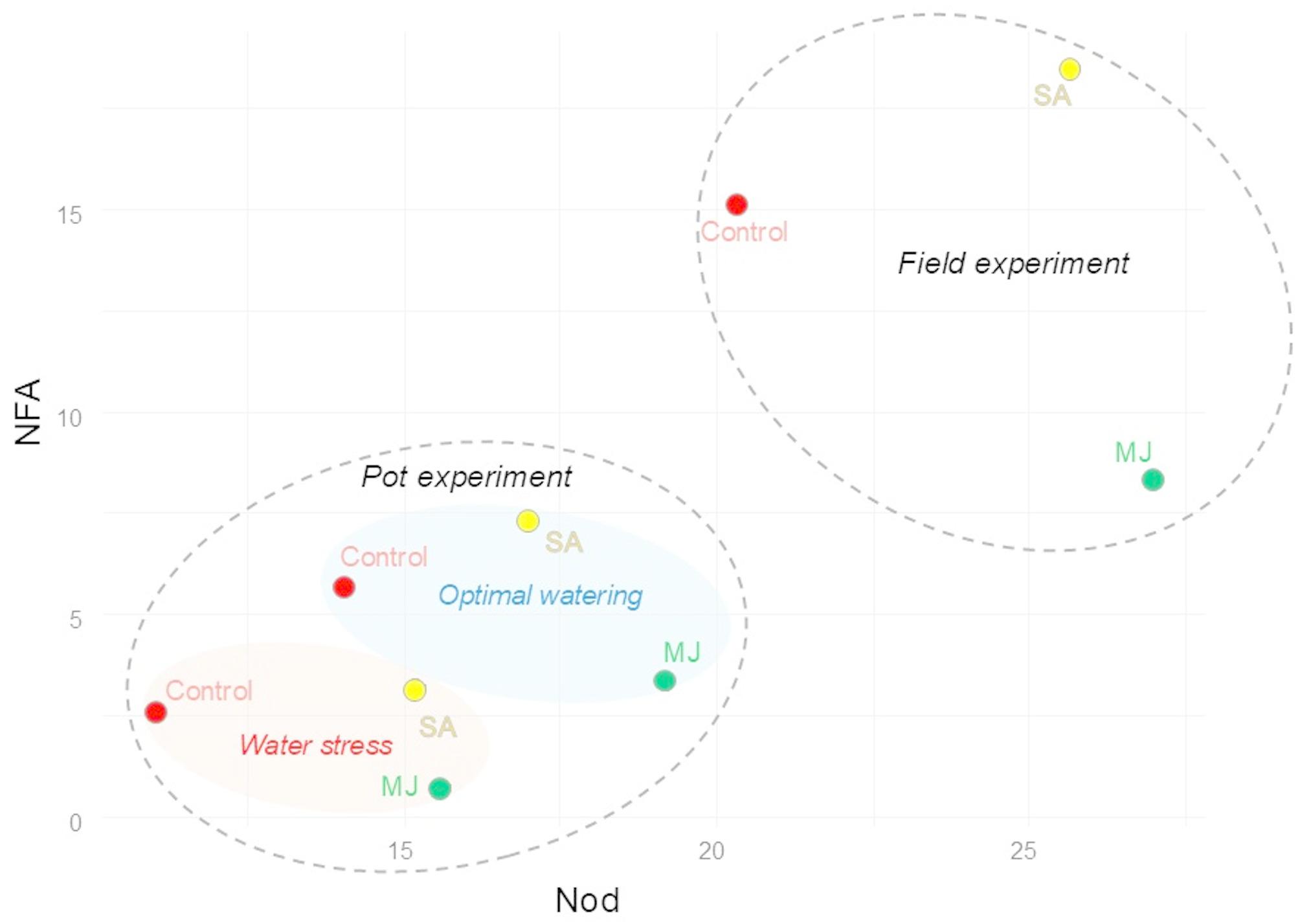
Scatterplot of Nod versus NFA values for experimental treatments in soybean under different conditions of water supply. Experimental cases are grouped into pot experiments and field experiments. Experimental treatments, MJ: methyl jasmonate (green), SA: salicylic acid (yellow), *Bradyrhizobium*: control (red)

The scatterplot of Nod and NFA provided further insights into the differentiation of experimental conditions (Fig. 11). Two major experimental groups were evident: field experiments, characterized by high Nod and NFA values, and pot experiments, which exhibited more diverse responses depending on water conditions. Within the pot experiments, cases under optimal watering formed a distinct cluster with intermediate Nod and NFA values, while cases under water stress were associated with the lowest Nod and NFA values.

The addition of MJ and SA treatments highlights their differential effects on these variables (Fig. 11). MJ treatments consistently exhibit moderate Nod and NFA values under both water conditions, while SA treatments enhance Nod and NFA, particularly under optimal watering. Control treatments show the lowest values under water stress and moderate performance under optimal watering.

The addition of MJ and SA treatments provides critical insights into their roles in modulating plant performance under different environmental conditions (Fig. 11). MJ appears to enhance stress resilience, while SA promotes nitrogen fixation under optimal conditions, offering potential strategies for improving plant productivity in varying water availability scenarios.

## Discussion

### Pro-antioxidant status in soybean nodules

The formation of a symbiotic interaction between macro- and microsymbionts, results in a cascade of biochemical reactions, some of which regulate the activity of the host plant’s protective systems with the participation of ROS [35]. It is known that ROS play a protective role in the functioning of plants under the influence of stress factors.

Under optimal growth conditions, the addition of GR to the *Bradyrhizobium* cultivation medium induces changes in the pro-antioxidant status of the soybean nodules. In the present study the SOD enzymatic complex reacted in various ways following inoculation with GR-modified *Bradyrhizobium*. In particular, under optimal irrigation conditions, SOD activity increased in the MJ variant but decreased in the SA variant; both variants also exhibited a decrease in H_2_O_2_ concentration and CAT activity, particularly the MJ variant. These changes in nodule antioxidant status could obviously activate a complex of biochemical reactions affecting the formation and functioning of the soybean symbiotic apparatus. In particular, while nodulation processes were stimulated in both the SА and MJ variants under optimal plant watering, nitrogen fixation activity increased in SA and decreased in MJ.

Under water stress conditions, both GR variants (SA and MJ) exhibited a different antioxidant status to those observed under optimal conditions, and both variants demonstrated elevated SOD and CAT enzymatic complex activity. However, the highest SOD activity was observed in the SA variant, and the highest CAT activity in the MJ variant. In the SA variant, the changes in SOD and CAT activity resulted in a fall in H_2_O_2_ content, thus relieving the effects of water stress; its level was also similar to that observed in soybean nodules under optimal irrigation. Hence, it appears that the inclusion of antioxidant enzymes in soybean nodules under the action of SA is able to neutralize the effects of oxidative stress caused by insufficient water supply. Co-inoculation of seeds with *Bradyrhizobium* and SA improved the symbiotic potential of soybean under the action of water stress. Our observations are in line with previous research indicating that SA can influence ROS production, as well as SOD, CAT and peroxidase activity and that these effects are accompanied by intracellular changes in plant metabolism and preadaptation to stress effects [7, 9].

During conditions of water stress, the MJ variant demonstrated increased activation of SOD and CAT in nodules, together with insignificant differences in H_2_O_2_ content compared to controls (i.e. without GRs). However, in the MJ variant, the H_2_O_2_ content under water stress was twice that observed under conditions of optimal irrigation. This indicates the development of oxidative processes and the inability of antioxidant enzymes to neutralize excessive production of H_2_O_2_ during water stress in the MJ variant. Interestingly, the MJ variant also demonstrated changes in the course of certain metabolic processes, which affected the functioning of the nitrogenase enzymatic complex and inhibited NFA in nodules both optimal watering and water stress.

Some reactions exhibited by legume plants to inoculation with rhizobia have appear to resemble those associated with infection by pathogenic microorganisms [35]. However, during nodule formation, these reactions have been found to regulate reproduction and metabolic activity in the symbiosomes, rather than inactivate the invading microorganism. Perhaps during the formation of symbiotic interaction, jasmonate activity may decrease H_2_O_2_ production in the nodules and alter CAT activity, decreasing or increasing it, according to optimal or insufficient water supply conditions; this would contrast with their typical role in pathogenesis.

The synthesis of CAT in peroxisomes is induced by the presence of H_2_O_2_, with a fairly high amount being required to initiate enzyme activity; in its absence, the enzyme activity may be low [36]. Cellular CAT is a synergist of SOD and prevents the accumulation of H_2_O_2_: and its presence inhibits SOD. The increase in CAT activity observed has been attributed under the action of stressors may be due to the activation of its latent forms or the synthesis of new molecules. It is known that the increase in endogenous synthesis of SA or MJ is accompanied by changes in the levels of phytohormonal balance, in particular, a decrease in cytokinins and auxins, together with an accumulation of abscisic acid, which has an important role in triggering plant defence reactions [3]. In addition, SA is known to regulate ROS production and antioxidant enzyme activity (SOD, CAT, peroxidase); both effects are accompanied by intracellular changes in plant metabolism and allow its preadaptation to stress [7, 9].

### Soybean symbiotic capacity

Rhizobial infection of legumes significantly modifies the metabolism of the host plant, which provides causes the creation of optimal conditions for infection and may be a prerequisite for the further effective functioning of the legume-rhizobial symbiosis [35]. Inoculating seeds with *Bradyrhizobium* modified with MJ has previously been found to stimulate nodulation processes and lower nitrogen fixation activity in soybeans at the early stages of symbiosis under optimal moisture conditions [37].

Similarly, our present findings show that inoculation with *Bradyrhizobium* containing MJ also reduces NFA in soybean nodules at later stages of symbiosis, i.e. from the formation of trifoliate leaves to flowering. A similar trend was recorded in the MJ variant used in the plot field experiment. The addition of MJ at a concentration of 0.75 µM to the *Bradyrhizobium* cultivation medium appears to induce a complex of biochemical processes that inhibition the functioning of the nitrogenase enzymatic complex in soybean nodules, at both the early and later stages of the soybean- *Bradyrhizobium* symbiosis. It is possible that changes observed in the functioning of the symbiotic apparatus under the influence of MJ may be associated with the formation of ineffective nodules on soybean roots, i.e. those which are unable to fix molecular nitrogen from the atmosphere.

There is currently is little data on jasmonate signal transduction and its integration with other metabolic pathways in the plant cell, in particular, pro-antioxidant systems. A few reports indicate that jasmonic acid signal transduction may be accompanied or implemented by ROS-dependent mechanisms [9]. However, this issue remains poorly understood, especially in the context of the its relationship with symbiotic signal transduction during the formation of soybean- *Bradyrhizobium* symbiosis.

The addition of SA at a concentration of 50 µM to the *Bradyrhizobium* medium intensified the nodulation processes in soybean under both optimal and insufficient moisture conditions. In addition, the addition appeared to improve the symbiotic apparatus of soybean, as evidenced by an increase in the NFA of nodules under different moisture conditions.

The inoculation of seeds with *Bradyrhizobium* and SA has previously been found to stimulate nodulation and nitrogen fixation in soybean nodules at the early stages of symbiosis formation under optimal irrigation conditions [37].

It has been noted that the very early stages of symbiotic interaction (pre-infection) are characterised by rapid activation of SA signaling transduction, which is subsequently suppressed; this promotes the growth of the infection thread and the formation of nodule primordia [19]. However, there is little information about the participation of SA in the functioning of symbiotic relationships at later stages, especially under the influence of insufficient moisture supply.

**Fig. 12:**
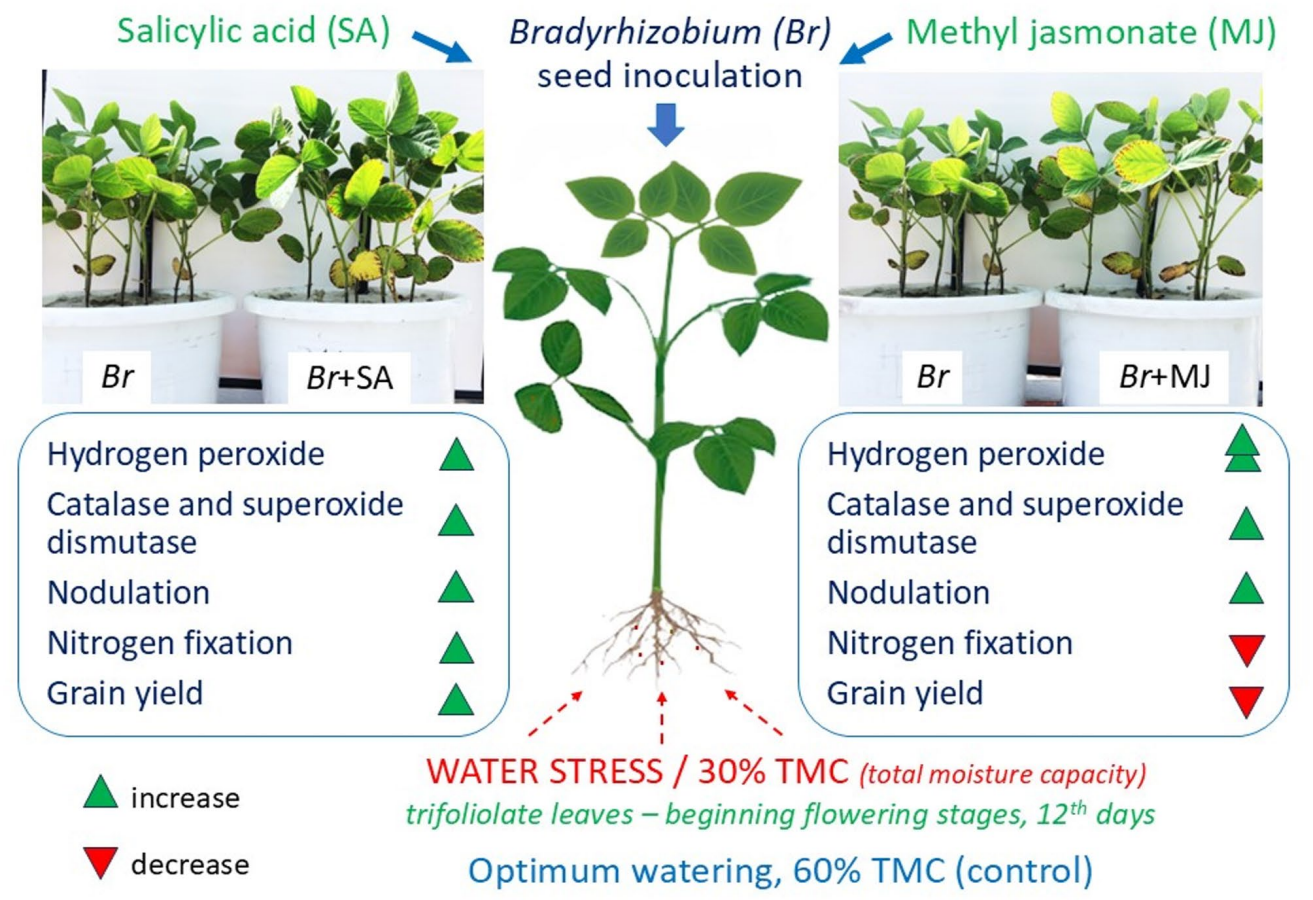
Schematic representation of the influence of SA and MJ in *Bradyrhizobium* culture medium on the formation of soybean symbiotic capacity. The direction of the arrows indicates the change in the levels of the specified parameter– increase (red) and decrease (green); double arrows of the same color emphasize significant changes in this parameter. Experimental treatments, MJ: Methyl Jasmonate, SA: Salicylic Acid, *Bradyrhizobium*: Control

The SA variant also demonstrated a positive effect on nitrogen fixation processes under field plot conditions, resulting in increased grain yield. Hence, supplementing the *Bradyrhizobium* culture medium with 50 µM SA appears to improve the efficiency of the soybean capacity under both optimal and insufficient moisture conditions (Fig. 12).

It is predicted that climate changes will be accompanied with increasing numbers of extreme weather events, including droughts and moisture deficits. It will also bring greater environmental pollution caused by the irrational use of agrochemicals such as nitrogen fertilizers. One way of avoiding such overuse would be to increase the nitrogen-fixing potential of legumes by optimising their symbiotic interaction with nodule bacteria.

The data presented in the article are in line with the European Commission policy for taking active measures to adapt to the coming impacts of climate change and preserving the natural environment. It examines how natural solutions (NBS) can be integrated into the large-scale biologization of leguminous agrotechnologies to ensure the realization of natural processes. More specifically, our study addresses the interaction between plant and rhizobium, which is a powerful factor in increasing the productivity of agrocenosis. By increasing such efficiency, it will be possible to ensure greater sustainability with regard to anthropogenic pressures and the provision of WBSRCE benefits, i.e. those related to water (W), biodiversity (B), ecosystem services (S), resilience (R), culture (C) and education (E). Our results describe a holistic approach to the effective use of ecological sources of nitrogen in the soil-plant-atmosphere system under climate change; it demonstrat how nature-based environmental solutions (NBS) can be combined with conventional elements of modern agricultural technologies to improve WBSRCE resources.

Our findings demonstrate that the use of highly-effective strains of Bradyrhizobium complaexed with growth-regulating compounds can increase the productivity of soybean-rhizobial symbioses and mitigate the effects of water stress. The resulting symbioses exhibit better antioxidative potential and a more optimal physiological state, thus increasing drought tolerance and improving the productive potential of soybeans under adverse growing conditions. Such strategies are important for supporting rational agriculture under conditions of modern climate change.

The presented study has important theoretical and applied significance, and makes a significant contribution to improving water stress resistance in legumes. Our data can be used in the development of new technologies for soybean cultivation, such as environmentally-safe and economically-beneficial fertilizers for legumes based on symbiotic bacteria.

## Conclusions

Adding 50 µM salicylic acid (SA) to the culture medium for effective *Bradyrhizobium* induces changes in antioxidant status under the influence of water stress; these changes are driven by the activation of SOD and CAT and reduction in H_2_O_2_ in nodules, which have positive effects on the symbiotic apparatus and soybean productivity. In contrast, the addition of 0.75 µM methyl jasmonate **(**MJ) to the *Bradyrhizobium* culture medium leads to excessive production of H_2_O_2_ and activation of CAT and SOD in soybean nodules under water stress. This is accompanied by both enhanced nodulation processes and decreased nitrogen fixation. Co-inoculation of *Bradyrhizobium* with MJ alters the metabolic processes that determine nitrogenase enzymatic complex functioning. It also inhibits the nitrogen-fixing activity of soybean nodules under optimal and insufficient water supply, which requires further research. The combined application of SА (50 µM) with *Bradyrhizobium* is an effective solution for activating the protective antioxidant properties of soybean plants, which helps to increase their symbiotic capacity and tolerance under water stress.

## Electronic supplementary material

Below is the link to the electronic supplementary material.

Supplementary Material 1

## Data Availability

Data is provided within the manuscript or supplementary information files.
