# Supplementary material for: Alleviation of water stress in soybean symbiosis by salicylic acid and methyl jasmonate-activated Bradyrhizobium
Source: BMC Plant Biol. 2025 Jul 3;25:862. doi: 10.1186/s12870-025-06806-1 (PMC12225050; doi:10.1186/s12870-025-06806-1)
Supplement: Supplementary file 1 — Supplementary Material 1 [file 12870_2025_6806_MOESM1_ESM.pdf]

Co-inoculation of Bradyrhizobium with methyl jasmonate and salicylic acid regulates the antioxidant and symbiotic capacity of soybean under water stress

Tetiana Nyzhnyk[1,2]*, Edyta Kiedrzyńska[2,3,4], Sergii Kots[1], Maciej Zalewski[2], Marcin Kiedrzyński[4*]

[1]Institute of Plant Physiology and Genetics of the National Academy of Sciences of Ukraine, Department of Symbiotic Nitrogen Fixation, Vasylkivska 31/17, 03022 Kyiv, Ukraine

[2] European Regional Centre for Ecohydrology of the Polish Academy of Sciences, Tylna 3, 90-364 Lodz, Poland

[3] University of Lodz, Faculty of Biology and Environmental Protection, UNESCO Chair on Ecohydrology and Applied Ecology, Banacha 12/16, 90-237 Lodz, Poland

[4] University of Lodz, Faculty of Biology and Environmental Protection, Department of Biogeography, Paleoecology and Nature Conservation, Banacha 1/3, 90-237 Lodz, Poland

*E-mail: tp_nyzhnyk@ukr.net; marcin.kiedrzynski@biol.uni.lodz.pl

CAT (Fig. 5)

Soybean	Experiment variant	Mean	Median	SD	Min	Max
Optimal_Water	Control_OW	3,4968	3,631	0,28	3,163	3,808
	SA_OW	3,2356	3,222	0,108	3,105	3,359
	MJ_OW	3,0674	3,089	0,125	2,89	3,21
Water_Stress	Control_WS	3,1258	3,155	0,134	2,965	3,264
	SA_WS	4,5162	4,476	0,244	4,233	4,877
	MJ_WS	4,835	4,884	0,094	4,703	4,918

SOD (Fig. 4)

Soybean	Experiment variant	Mean	Median	SD	Min	Max
Optimal_Water	Control_OW	84,3864	82,51	5,517	78,37	91,16
	SA_OW	69,894	67,84	5,93	64,53	78,11
	MJ_OW	95,2854	98,97	17,496	73,73	117,327
Water_Stress	Control_WS	91,732	89,42	11,851	79,09	111,31
	SA_WS	110,7262	108,307	11,302	98,701	126,24
	MJ_WS	105,1438	104,95	12,009	88,61	121,479

H202 (Fig. 3)

Soybean	Experiment variant	Mean	Median	SD	Min	Max
Optimal_Water	Control_OW	37,91	38,27	1,795	35,45	39,92
	SA_OW	29,336	28,51	2,76	26,5	33,67
	MJ_OW	23,486	23,45	1,788	20,903	25,52
Water_Stress	Control_WS	73,851	74,21	3,273	69,75	77,75
	SA_WS	33,611	33,015	2,173	30,75	36,47
	MJ_WS	69,716	69,37	1,747	67,56	72,22

Nod (Fig. 7)

Soybean	Experiment variant	Mean	Median	SD	Min	Max
Optimal_Water	Control_OW	14,02	14	1,565	12	16
	SA_OW	17	18	2	14	19
	MJ_OW	19,2	19	1,923	17	22
Water_Stress	Control_WS	11	11	1,581	9	13
	SA_WS	15,2	15	3,033	11	19
	MJ_WS	15,6	17	3,435	11	19

Massa_Nod (Fig. 8)

Soybean	Experiment variant	Mean	Median	SD	Min	Max
Optimal_Water	Control_OW	0,445	0,445	0,038	0,401	0,504
	SA_OW	0,39	0,354	0,087	0,31	0,537
	MJ_OW	0,281	0,249	0,108	0,182	0,466
Water_Stress	Control_WS	0,242	0,249	0,035	0,184	0,275
	SA_WS	0,264	0,266	0,015	0,242	0,286
	MJ_WS	0,249	0,248	0,028	0,226	0,296

NFA (Fig. 9)

Soybean	Experiment variant	Mean	Median	SD	Min	Max
Optimal_Water	Control_F	5,649	5,878	0,866	4,489	6,745
	SA_F	7,3	7,367	1,095	5,912	8,552
	MJ_F	3,307	3,202	0,807	2,334	4,334
Water_Stress	Control_WS	2,572	2,667	0,285	2,173	2,873
	SA_WS	3,178	3,272	0,451	2,544	3,622
	MJ_WS	0,68	0,681	0,191	0,433	0,882

NFA_Field (Fig. 10)

Soybean	Experiment variant	Mean	Median	SD	Min	Max
**Fild**	Control_F	15,097	14,305	2,434	12,67	18,87
	SA_F	18,479	18,768	1,407	16,13	19,76
	MJ_F	8,3	8,35	1,699	6,39	10,37

Nod_Field (Fig. 10)

Soybean	Experiment variant	Mean	Median	SD	Min	Max
**Fild**	Control_F	20,333	20	2,422	18	24
	SA_F	25,666	25,5	1,632	24	28
	MJ_F	27	28	4,647	21	33

Massa_Nod_Field (Fig. 10)

Soybean	Experiment variant	Mean	Median	SD	Min	Max
**Fild**	Control_F	0,513	0,515	0,136	0,31	0,7
	SA_F	0,503	0,515	0,053	0,41	0,56
	MJ_F	0,465	0,45	0,072	0,4	0,59

Grains_Mass (Fig. 11)

Soybean	Experiment variant	Mean	Median	SD	Min	Max
**Fild**	Control_F	2,105	2,07	0,274	1,8	2,57
	SA_F	2,441	2,48	0,262	2	2,76
	MJ_F	1,918	1,87	0,318	1,58	2,42
